# Set-up and input dataset files of the Delft3d model for hydrodynamic modelling considering wind, waves, tides and currents through multidomain grids

**DOI:** 10.1016/j.dib.2019.104921

**Published:** 2019-12-09

**Authors:** Juan Gabriel Rueda-Bayona, Andrés F. Osorio, Andrés Guzmán

**Affiliations:** a Universidad Militar Nueva Granada. Engineering Faculty. Civil Engineering, Water and Energy (AyE) Research Group, Bogotá: Carrera 11 No.101- 80, Colombia; b Universidad Nacional de Colombia, Departamento de Geociencias y Medio Ambiente, Grupo de Investigación OCEÁNICOS, Carrera 80 No 65-223, Medellín, Colombia; c Universidad del Norte, Department of Civil and Environmental Engineering, Research group for structures and geotechniques (GIEG), Área Metropolitana de Barranquilla, Km 5 via Puerto Colombia, Bloque K, 8-33K, Barranquilla, Colombia

**Keywords:** Numerical modelling, Simulation, Hydrodynamic, Delft3D, Waves, Wind, Tides, Currents

## Abstract

This article contains the set-up and input files of the implementation of Delft3D model to determine extreme hydrodynamic forces performed in Rueda-Bayona et al. [1]. The model was configured with a multidomain grid using double-way communication between the hydrodynamic and wave module. The multidomain grids solve faster than single and nested grids because require less grid points to calculate. Also, the double-way communication between the hydrodynamic and wave modules allows to consider the non-linear interactions of wind, waves, tides and currents. Because there are no modelling examples related to multidomain grids in the open access official web site of Delft3d model, this data contributes to increase the availability information of this necessity. Finally, the files of this article are ready to be run in the Delft3D model to perform a sensitivity test recommended in Rueda-Bayona et al. [1].

**Table:** Specifications Table

Subject	Engineering
Specific subject area	Ocean engineering
Type of data	Set-up and input files to run the Delft3D model.
How data were acquired	Implementation and numerical simulation in a Windows Core i7 computer with x64 bits.
Data format	Raw
Parameters for data collection	The considered conditions for using the input and set-up files are described in Rueda-Bayona et al. [1,2].
Description of data collection	The set-up files where configured, implemented and run considering numerical restrictions and know-how of the Delft3D model [3,4].
Data source location	The study area is limited by the following coordinates: X1 = −72.585997° W, Y1 = 12.5° N X2 = −72.213488, Y2 = 12.065316° N X3 = −72.585997° W, Y3 = 12.065316° N X4 = −72.213488° W, Y4 = 12.5° N
Data accessibility	Supplementary material alongside the online version of this data article.
Related research article	J.. Rueda-Bayona, A. Osorio-Arias, A. Guzmán, G. Rivillas-Ospina, Alternative Method to Determine Extreme Hydrodynamic Forces with Data Limitations for Offshore Engineering, J. Waterw. Port, Coastal, Ocean Eng. 145 (2018) 05018010. https://doi.org/10.1061/(asceww.1943-5460.0000499) [1].

**Table:** 

**Value of Data** • The data is useful to calculate hydrodynamics in study zones with data limitations. • The data (set-up and input files) allow to estimate hydrodynamic forces and currents power densities potential in the Guajira, Colombia. • This data contains a numerical study case (set-up and input files) performed through the Delf3D model that can be considered as examples of numerical multidomain modelling. • This data can be used as tool to perform preliminary feasibility assessment of offshore projects.

## 1 Data

**Fig. 1:**
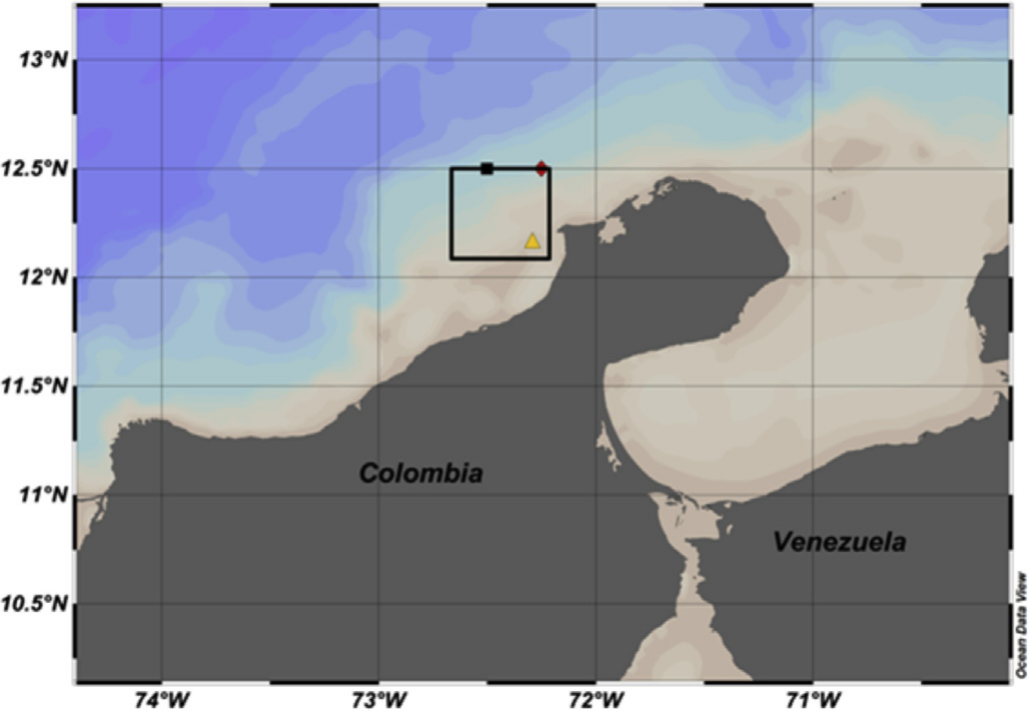
Study area and Outer Grid of the multidomain model (black polygon).

The dataset gathers the input and set-up files of a study case modelled through the Delft3D model. The data allows to model a multidomain grid with double-way communication in an offshore location of the Guajira – Colombia (Fig. 1). Also, this data can be considered as a reference to implement multidomain grid modelling for others study cases.

The input data contains information of atmosphere (relative humidity, air temperature, cloud coverage, solar radiation, wind) extracted from NARR-NOAA database [5], water levels calculated through the GRENOBLE model [6], bathymetry from ETOPO database [7], surface salinity and temperature of the study area derived from World Ocean Atlas database (www.nodc.noaa.gov) [8]. The set-up files contain model parameters that specify the boundary conditions, grid geometry, govern equations to be solve, and the coordinates of the monitoring observation points. The wave data utilized as input information was extracted from the database provided by Oceanicos-UNAL et al. [9] and related to the research of A.F. Osorio et al. [10].

The data is gathered and stored within a compressed folder named as Multi_domain_2004_all_forces.zip. The Multi_domain_2004_all_forces folder contains the input and setup files of the Delf3d model mentioned above; the guajira.ddb file allows to connect the outer and inner grid. The dataset can be downloaded directly from the online version of this data article.

## 2 Experimental design, materials, and methods

The study area of the multidomain modelling [1] is shown in Fig. 1, where the black square, red rhomboid and yellow triangle symbols, indicate the temperature-salinity input data, waves input data and numerical monitoring point respectively (Fig. 2 of [1]).

The study area is considered as strategic (Fig. 1) because there were identified the highest wind speed and wind power density potential in Colombia according to the results revealed in Rueda-Bayona et al. [11].

The dataset of this article is in ASCII file format, and is organized and described as follows:

### 2.1 Input data

• Outer bathymetry: outside.dep.
• Inner bathymetry: inside.dep.
• Boundary definition file (Flow module): 2004.bnd
• Time-series flow conditions (Flow module): 2004.bct.
• Transport conditions (Flow module): 2004.bcc.
• Bottom roughness file (Flow module): Chezy_5_60.rgh
• Heat flux model data (Flow module): 2004.tem.
• Wind data (Flow module): 2004.wnd.
• Wave boundary condition: TPAR.bnd.

### 2.2 Set-up data

• Outer Grid (Flow module): outside_Guajira_2004.grd.
• Inner Grid (Flow module): inside_Guajira_2004.grd.
• Wave module grid: outside_swan.grd.
• Outer enclosure grid: outside_Guajira_2004.enc.
• Inner enclosure grid: inside_Guajira_2004.enc.
• Outer grid observation points: outside_puntos.obs.
• Inner grid observation points: windmill.obs.

The data related to the atmosphere information (2004.tem, 2004.wnd) were processed through MATLAB (www.mathworks.com) language with the same methodology recommended in the data article of Rueda-Bayona et al. [12]. The bathymetry, geometry and monitoring point information (outside.dep., inside.dep, Chezy_5_60.rgh, outside_Guajira_2004.grd., inside_Guajira_2004.grd., outside_Guajira_2004.enc., inside_Guajira_2004.enc., outside_puntos.obs., windmill.obs.) were created through the RGFGRID and QUICKIN tools of the Delft3D model. The Boundary definition, Time-series flow conditions, Transport conditions, were generated with the graphical user interface (GUI) of the flow module and verified though EXCEL spreadsheets. Finally, the Wave boundary condition data (TPAR.bnd.) was processed in MATLAB.
